# Injuries of the Head

**Published:** 1829-07-01

**Authors:** 


### BATH HOSPITAL.

Several instructive cases of injury of the head have been reported from this hospital in the Provincial Medical Gazette; passing over the first, a fatal and not very unusual instance of hernia cerebri, in consequence of wound of the dura mater, we are led to take notice of the following, because it involves a practical question in surgery, or rather illustrates a position on which we have often dwelt.

I. Compound Fracture and Depression of the Skull—Operation—Recovery.

William Wilmott, 60 years of age, fell from a height of fourteen feet in a quarry, and struck his head upon the angle of a block of stone. He was admitted into the hospital shortly afterwards perfectly sensible, with his skin cold, and the pulse small and weak. At the upper and posterior part of the parietal bone was a small wound which bled freely and communicated with the bone, which was fractured and depressed. An oval piece, about an inch and a half long and half an inch in breadth, was indented, one side remaining on a level with the rest of the skull, the other being depressed a quarter of an inch. The depressed part was so firmly wedged down, that it could not be raised until the level edge was completely divided by Hey's saw, when the bone was removed, and, as usually happens, the fracture proving much more extensive in the internal table than the external, several other fragments were taken away. The dura mater was unhurt, and no extravasation existed between it and the bone.

The patient passed a restless night, and was purged and bled to eighteen ounces next day. On the 20th of August, (the accident happened on the 18th) there was a copious discharge of watery fluid from the surface of the dura mater, but the wound had in great measure closed by adhesion. There was a good deal of pain in the back of the head, intolerantia lucis, pulse 70, tongue moist. He passed a restless night, and on the 21st, was bled again to sixteen ounces. Want of sleep, and pain in the head, with a pulse rather slow than quick, viz, from 58 to 64 in the minute, continued to be the more prominent symptoms, and on the 25th, he lost six ounces more blood. A thin, clear discharge flowed for some time from the surface of the dura mater, but the wound healed slowly, and the patient was discharged in good general health, on the 9th of October. The pulse at this time was still slow, and he suffered from giddiness in the head and dimness of vision.

This case is really a fine illustration of Sir Astley Cooper's precept of operating in *compound* fracture, with depression of the cranium, whether there be present symptoms or not. Of course we cannot pretend to affirm, in a positive manner, that under other treatment than that which was adopted a different result must necessarily have followed, but we do believe that in all probability it would. Cases apparently similar have occurred where the broken bone was not interfered with, and the consequence has been, the supervention of fatal inflammation and suppuration on the surface of the dura mater, or in the brain, as, for instance, in the case reported at page 234. Cases have also occurred where the operation which was neglected or rejected in the first instance, has perforce been had recourse to at the secondary period when inflammation has come on, sometimes with success, oftener, much oftener without it. On these and on other accounts, we think that Mr. Norman, under whose care the present case was, acted scientifically and judiciously in applying the saw, and raising the depression, even although it produced no symptoms of pressure at the time. With respect to the giddiness in the head and the weakness of vision that remained when the man was apparently recovered, we believe that these dregs, if so we may term them, of affections of the head after injury inflicted, are by no means uncommon. It is not at all unusual to find a patient complaining for months and months of violent pain in the head, "a solitary symptom after all other symptoms have vanished."

II. Fracture of the Cranium with slight Depression—Wound of the Meningea Media — Operation — Death.

We are tempted to take notice of this case also, because it has a practical bearing, and is one that will every now and then be met with by the surgeon.

A lad of 16, was brought into the hospital under the charge of Mr. W. Brown, on the 5th of November, having fallen on his head from a hay-loft fourteen feet high. Immediately above the ear was a very slight scalp wound, and although he was stunned, he was far from completely insensible, desiring the attendants not to cut his clothes in removing them. By the end of an hour, a material change had taken place; he had vomited several times—his respiration was stertorous, though not in any great degree—his pulse was weak, and had become more frequent—the pupil of the right eye dilated, that of the left in its natural state, and acting freely on the access of light—he was much less sensible, but still expressed pain, when firm pressure was made in the seat of injury. Mr. Brown, who saw him two hours after the accident, divided the scalp by a T shaped incision immediately above the ear — exposed the bone — found a fissure extending from the vertex through the lateral and posterior parts of the parietal and temporal bones towards the basis, with a trifling depression below—applied the trephine and Hey's saw, and removed the depressed piece.

The finger now passed into an extensive, collection of coagulated blood, beneath which the dura mater could be felt deeply depressed and detached from the bone in a circle of two or three inches in diameter. The coagulum was removed to the amount of four or five ounces, and the dura mater seen to be uninjured. As fast, however, as it was removed it re-accumulated with rapidity. The source of the hæmorrhage was discovered to be a tolerably large branch of the meningeal artery, pouring out blood with a vigorous jet, some way within the anterior margin of the bone. After some time had been allowed to pass in the fruitless expectation that the bleeding would cease, a tenaculum was carefully passed under the bleeding point, so as not to perforate the whole thickness of the dura mater, and the vessel then secured by ligature. The surface of the dura mater which had not at all risen was next cleared from blood; the integuments closed by a few points of suture, the head turned to the injured side, and covered with cloths kept wet by cold water. He lost much blood during the operation and was rather more sensible after it, whilst the states of the pupil, respiration, and pulse had also undergone an improvement. In the evening re-action took place, and six ounces of blood were abstracted; at eight, p. m. the dura mater had regained its natural elevation, the breathing was easy, the pulse 96. He was purged, but next morning the pupil was more dilated and immoveable, the pulse 132, the patient restless. Another purgative was given, and after its operation he seemed to be relieved. However, no material amelioration ensued; the insensibility continued, as did the restlessness; the pupils, and especially the right, were dilated; the pulse was rapid, always above 130, and early in the morning of the 11th, he died.

*Dissection.* The fracture extended from the upper and posterior part of the right parietal bone to the basis cranii, passing through the squamous part of the temporal, running round the petrous portion, which was loose, and terminating in the body of the sphenoid bone. The dura mater, where it had been detached from the bone, "was alive," and covered by a thin but very firm layer of pale red coagulum. The ligature, which was loose in the wound, had been applied to one of the principal divisions of the meningea media, and had not been passed through the whole thickness of the dura mater. The principal injury to the brain was on the side opposite to the fracture, consisting of contusion with effusion of blood under the pia mater, and into the superficial parts of the brain at several points. There were few traces of inflammation, and those almost wholly confined to the upper part of the hemispheres, where the arachnoid was opaque, and the vessels of the pia mater injected in patches of small extent.

Here also we think that Mr. Norman acted very properly in applying the trephine, even though success did not consecrate the operation. A boy receives a fall upon the head, which produces a scalp-wound and *does not completely* stun him. In the course, however, of an hour, he sinks into a condition of total, or almost total, insensibility, accompanied with stertor and the other symptoms said to indicate pressure on the brain. Now what state of things can such a series of phenomena point out? Concussion? No, for that would not increase, but be greatest at the time of the accident. Compression from broken and depressed bone? Surely something more, for that too would be an injury acting at once, and not supervening in a gradual manner. The symptoms are those of pressure, and the pressure is evidently produced by extravasation of blood, *because* it increases gradatim and before inflammation could possibly be set up, just as the blood would pour out from a ruptured vessel. Under these circumstances the surgeon finds a scalp-wound, enlarges it, and discovers a fissure. Now we do say, that in such a combination of things, not only is the surgeon right in trephining, but, cæteris paribus, he is *bound* to do it, for although the extravasation *may* not be there, the probabilities are strongly that it will. In this sort of case it seems to us that the surgeon then ought to trephine, and that whether he finds the extravasation, or whether he does not, he has done his duty. The case, however, is materially altered, when the fissure or fracture of the skull is absent, the other symptoms and the order of their occurrence being still the same. We know that there is pressure, that that pressure is probably from extravasation, but we have not the fracture to guide us to the spot, we want the characters graven, as it were, on the tablet of the skull, telling us "here ye may search and find." There may be (and generally is) a scalp wound, the bone may even be denuded, and yet the effusion may be quite on the opposite side of the skull, and trephining, if performed, must be worse than useless. In such a case, and unhappily it is not uncommon, a fearful uncertainty prevails, and a trying question there is for the surgeon to determine.

